# YAP-Dependent BiP Induction Is Involved in Nicotine-Mediated Oral Cancer Malignancy

**DOI:** 10.3390/cells10082080

**Published:** 2021-08-13

**Authors:** Chu-Yen Chien, Ying-Chen Chen, Chia-Chen Hsu, Yu-Ting Chou, Shine-Gwo Shiah, Shyun-Yeu Liu, Alexander Cheng-Ting Hsieh, Ching-Yu Yen, Chien-Hsing Lee, Yi-Shing Shieh

**Affiliations:** 1 Graduate Institute of Medical Sciences, National Defense Medical Center, Taipei 114, Taiwan; pooh.chien@gmail.com (C.-Y.C.); because0517@gmail.com (C.-C.H.); 2 Molecular and Cell Biology, Taiwan International Graduate Program, Academia Sinica and Graduate Institute of Life Science, National Defense Medical Center, Taipei 114, Taiwan; jamiechen1027@gmail.com; 3 Institute of Biotechnology, National Tsing Hua University, Hsinchu 300, Taiwan; ytchou@life.nthu.edu.tw; 4 National Institute of Cancer Research, National Health Research Institutes, Miaoli 350, Taiwan; davidssg@nhri.edu.tw; 5 Department of Oral and Maxillofacial Surgery, Chi Mei Medical Center, Tainan 710, Taiwan; syliu@mail.chimei.org.tw; 6 School of Traditional Chinese Medicine, Chang Gung University, Taoyuan 333, Taiwan; alexsctshieh@gmail.com; 7 School of Dentistry, Taipei Medical University, Taipei 110, Taiwan; 8 Division of Endocrinology and Metabolism, Department of Internal Medicine, Tri-Service General Hospital, National Defense Medical Center, Taipei 114, Taiwan; 9 Department and Graduate Institute of Biochemistry, National Defense Medical Center, Taipei 114, Taiwan; 10 Department of Dentistry, Tri-Service General Hospital, National Defense Medical Center, Taipei 114, Taiwan

**Keywords:** chaperons, cigarette smoking, nicotinic acetylcholine receptors, oncogenes, oral squamous cell carcinoma

## Abstract

Cigarette smoking is a significant risk factor for the development and progression of oral cancer. Previous studies have reported an association between nicotine and malignancy in oral cancer. Recent studies have also demonstrated that nicotine can induce endoplasmic reticulum (ER) stress in tumor cells. Binding immunoglobulin protein (BiP) acts as a master regulator of ER stress and is frequently overexpressed in oral cancer cell lines and tissues. However, the effect of nicotine on BiP in oral cancer is unknown. Therefore, this study aimed to evaluate the role of BiP and its underlying regulatory mechanisms in nicotine-induced oral cancer progression. Our results showed that nicotine significantly induced the expression of BiP in time- and dose-dependent manners in oral squamous cell carcinoma (OSCC) cells. In addition, BiP was involved in nicotine-mediated OSCC malignancy, and depletion of BiP expression remarkably suppressed nicotine-induced malignant behaviors, including epithelial–mesenchymal transition (EMT) change, migration, and invasion. In vivo, BiP silencing abrogated nicotine-induced tumor growth and EMT switch in nude mice. Moreover, nicotine stimulated BiP expression through the activation of the YAP-TEAD transcriptional complex. Mechanistically, we observed that nicotine regulated YAP nuclear translocation and its interaction with TEAD through α7-nAChR-Akt signaling, subsequently resulting in increased TEAD occupancy on the HSPA5 promoter and elevated promoter activity. These observations suggest that BiP is involved in nicotine-induced oral cancer malignancy and may have therapeutic potential in tobacco-related oral cancer.

## 1. Introduction

Oral cancer is the sixth most common cancer, accounting for 5% of all cancer cases worldwide [1,2]. Oral squamous cell carcinoma (OSCC) represents over 90% of all pathological types of oral cancer, and it is a major cause of cancer morbidity and mortality [1]. Treatment for oral cancer includes surgical eradication, radiotherapy, chemotherapy, and targeted therapy [3]. Despite advances in diagnosis and therapy, the 5-year survival rate for patients with oral cancer has not significantly improved and remains at 65% [4]. Therefore, it is important to understand the underlying mechanisms of oral cancer progression. In Southeast Asian countries, oral cancer is a major epidemiological concern due to the habits of betel quid chewing, cigarette smoking, and alcohol drinking [2]. Among them, cigarette smoking is a significant etiological factor associated with the development and progression of oral cancer. More than 7000 components, of which at least 70 are carcinogens, have been identified in tobacco smoke [5]. Previous studies have demonstrated that nicotine, the major addictive constituent of cigarettes, can increase cell proliferation, epithelial–mesenchymal transition (EMT) change, metastasis, and chemoresistance in oral cancer [6,7,8]. However, the underlying mechanisms involved in nicotine-mediated oral cancer progression are not fully understood.

Recently, several studies have demonstrated nicotine-induced endoplasmic reticulum (ER) stress in both malignant and non-malignant cells, including rat placental trophoblast giant cells, normal lung cells, and lung cancer cells [9,10]. Microenvironmental and intracellular stresses including hypoxia, hypoglycemia, acidosis as well as calcium and redox imbalance disturb ER function and may result in the accumulation of misfolded and unfolded proteins, thereby causing ER stress [11]. To overcome ER stress, eukaryotic cells activate homeostatic responses, collectively termed the unfolded protein response (UPR). Increasing evidence has demonstrated that ER stress and UPR components are involved in tumor malignancy [12,13]. Binding immunoglobulin protein (BiP) belongs to the heat shock protein 70 (HSP70) family and is composed of a conserved ATPase domain and peptide-binding domain. It is normally expressed at low basal levels in adult organs and is critical for protein folding. However, the hyperexpression of BiP has been observed in tumor tissues and implicated in tumor proliferation, invasion, metastasis, and resistance to cancer therapies [14,15,16]. In vitro study has demonstrated higher levels of BiP in oral cancer cell lines compared to normal oral keratinocytes [17]. In addition, tissues from patients with OSCC have also shown remarkably higher expression of BiP compared to normal oral tissues [18]. Interestingly, in HepG2 human hepatoma cells and BeWo human placental trophoblastic cancer cells, nicotine exposure has been reported to increase the expression of BiP [19,20]. However, the effect of nicotine on BiP in oral cancer is still unknown.

Nicotine has been demonstrated to have tumorigenic and tumor-promoting activities mainly through the activation of nicotinic acetylcholine receptors (nAChRs) in several types of cancer, including oral cancer [21]. nAChRs are a family of ligand-gated cation channels that are widely expressed in many tissues, including oral epithelial cells [22]. The homopentamer α7-nAChR, a subtype of nAChR, has been highly associated with proliferation, angiogenesis, and metastasis in cancer [23]. In oral cancer, nicotine has been shown to stimulate cell proliferation and inhibit apoptosis through α7-nAChR [6,7,24,25]. A recent study showed that α7-nAChR signaling was involved in nicotine-regulated UPR activation in rat and human pancreatic β-cell lines [26]. However, the role of α7-nAChR in the regulation of UPR components, especially BiP, upon nicotine exposure in oral cancer is still under investigation.

In the present study, we aimed to investigate the potential role of the ER stress-responsive component, BiP, and its underlying regulatory mechanisms in nicotine-induced oral cancer malignancy. Our results demonstrated that BiP was involved in the pro-malignant effect of nicotine on oral cancer. In addition, the YAP-TEAD transcriptional complex acted as a downstream effector of the α7-nAChR-Akt axis in the induction of BiP expression.

## 2. Materials and Methods

### 2.1. Cell Line and Cell Culture

The human OSCC cell lines, OE and SAS, were grown in RPMI medium (GIBCO, Eggenstein, Germany) supplemented with 10% fetal bovine serum (FBS) (GIBCO, Eggenstein, Germany) and 1% Penicillin-Streptomycin-Amphotericin B (PSA) (Biological industries, Cromwell, CT, USA). The OE (OECM-1) cells were obtained from Merck Millipore, Darmstadt, Germany. The SAS cells were kindly provided by Dr. Michael Hsiao at Genomics Research Center, Academia Sinica, Taipei, Taiwan.

### 2.2. Drugs and Antibodies

Nicotine (Sigma-Aldrich, St. Louis, MO, USA) was dissolved in ethanol. The primary antibodies used were as follows: BiP (610978), E-cadherin (610182), and ZO-1 (610966) from BD Biosciences, San Jose, CA, USA; fibronectin (ab32419) from Abcam, Cambridge, MA, USA; occludin (#71–1500) from Thermo Fisher, Pittsburgh, PA, USA; α7-nAChR (TA321939) from OriGene, Rockville, MD, USA; phospho-YAP (Ser127) (#13008), YAP (#14074), TEAD (#13295), phospho-Akt (Ser473) (#9271S), Akt (#9272S), vimentin (#5741S) and GAPDH (#5174S) from Cell signaling, Beverly, MA, USA. Horseradish peroxidase (HRP)-conjugated secondary antibodies were purchased from Jackson ImmunoResearch Laboratories Inc., West Grove, PA, USA.

### 2.3. RNA Interference

siRNAs were synthesized by Dharmacon (Lafayette, CO, USA). The siRNA sequences used for targeting BiP and YAP were 5′-CCACCAAGAUGCUGACAUU-3′ and 5′-CCACCAAGCUAGAUAAAGA-3′, respectively. The siRNA used for targeting α7-nAChR was SMARTpool siRNA (a mixture of four individual siRNAs provided as a single reagent). The sequence for non-targeting siRNA was 5′-UAGCGACUAAACACAUCAA-3′. Transfection of siRNA (20 nM) into cells was performed using GenMute siRNA transfection reagent (SignaGen Laboratories, Ijamsville, MD, USA) following the manufacturer’s instructions.

### 2.4. RNA Extraction and Quantitative Real-Time PCR (RT-PCR)

Total RNA was extracted using TRIzol reagent (Invitrogen, Carlsbad, CA, USA) and cDNA was generated by reverse transcription of total RNA using a High-Capacity cDNA Synthesis kit (Applied Biosystems, Carlsbad, CA, USA) according to the manufacturer’s instructions. RNA was quantified using a NanoDrop Spectrophotometer (NanoDrop, Wilmington, DE, USA). The expressions of related genes in our study were analyzed using quantitative RT-PCR. Quantitative RT-PCR was performed on a Roche LightCycler 480 system with SYBR Green I Master mix (Roche, Indianapolis, IN, USA). The conditions were as follows: an initial heat denaturation at 95 °C for 2 min, followed by 40 cycles at 95 °C for 5 s and 60 °C for 20 s. Glyceraldehyde 3-phosphate dehydrogenase (GAPDH) was used as an internal control. The primer sequences are listed in Table 1.

### 2.5. Scratch Wound Healing Assay

Cell migration ability was determined using a wound-healing assay. Cells were plated and grown to confluence in six-well plates. The confluent cell monolayer of each well was scratched using a sterile micropipette tip to create a wound. After washing with PBS to remove floating debris, the cells were cultured for an additional 18 h. Closure of the wounded areas was observed using an inverted microscope and photographed at 0 and 18 h. ImageJ software was used to quantify the wound area. The migratory ability was calculated by the area reduction at 18 h compared to the wound area at 0 h.

### 2.6. Transwell Invasion Assay

Cell invasion ability was analyzed using a Transwell assay, which was carried out in 24-well plates using Transwell chambers with an 8-μm pore size (Millipore, Billerica, MA, USA) pre-coated with Matrigel (Corning Inc., Corning, NY, USA). Cells were seeded at 2.5 × 10^4^ in the upper chamber of the insert. After 24 h incubation, the invaded cells on the bottom side of the membrane were fixed with 100% methanol, stained with propidium iodide (PI), and photographed. The number of invaded cells per microscopic field was determined using ImageJ software.

### 2.7. Co-Immunoprecipitation

Co-immunoprecipitation was performed to analyze interactions between YAP and TEAD proteins. In brief, whole-cell lysates were mixed with the respective antibodies or IgG control overnight at 4 °C. Protein-antibody complexes were incubated with protein G sepharose beads (GE Healthcare, Piscataway, NJ, USA) on a turning wheel for 4 h at 4 °C and immune complexes were washed with PBS and boiled in SDS sample buffer for 10 min. The eluted proteins were subsequently analyzed by Western blot analysis.

### 2.8. Chromatin Immunoprecipitation

Chromatin immunoprecipitation (ChIP) was performed to analyze interactions between the HSPA5 promoter and TEAD protein. The ChIP assay was carried out using a Pierce magnetic ChIP kit (Thermo Fisher, Pittsburgh, PA, USA) according to the manufacturer’s protocols. Briefly, cells were cross-linked in 1% formaldehyde for 10 min, and glycine was added to the cells for 5 min at room temperature to terminate the cross-linking reaction. Subsequently, chromatin was sheared to fragments by sonication, and the cellular debris was removed by centrifugation. Ten microliters of the cell lysate served as the input control. The remaining samples were divided into two groups followed by incubation with anti-TEAD and non-specific rabbit IgG with rotation at 4 °C overnight. The immunocomplexes were precleared with protein A/G magnetic beads for 2 h at 4 °C with mixing. Chromatin was eluted by elution buffer, and crosslinks between protein and DNA were reversed by adding 6 μL of 5 M NaCl and 2 μL of 20 mg/mL Proteinase K at 65 °C for 1.5 h. Finally, DNA was recovered using a spin column. Precipitated DNA was used as the template for PCR. The sequences of the PCR primers used for amplifying the HSPA5 promoter region containing the TEAD binding site were as follows: forward 5′-GGCATTATCAAGACGATTTTCGC-3′ and reverse 5′-GGTTATCATTTACGGGGCTTTC-3′.

### 2.9. Luciferase Dual Assay

Transcriptional activity of the HSPA5 promoter was measured using a luciferase dual assay system. The HSPA5 promoter region containing the TEAD consensus binding motif was amplified by PCR using the designed primers, forward 5′-GGACTAGTCCACGGTAGGCTTTCAG-3′ and reverse 5′-CGCGGATCCCTTGCCAGCCAGTTG-3′, and cloned into the pMCS-Cypridina luciferase reporter vector (Thermo Fisher, Pittsburgh, PA, USA). The pTK-Red Firefly luciferase vector (Thermo Fisher, Pittsburgh, PA, USA) was used as an internal control. Polyjet transfection reagent (SignaGen Laboratories, Ijamsville, MD, USA) was used for co-transfection of the two plasmids. Luciferase activity was detected using a luciferase dual assay kit (Thermo Fisher, Pittsburgh, PA, USA) according to the manufacturer’s instructions. Firefly luciferase activity (encoded by the control plasmid) was used to normalize Cypridina luciferase activity (encoded by the experimental plasmid).

### 2.10. Immunohistochemistry

Immunohistochemical staining was performed using a Novolink polymer detection system kit (Leica Biosystems Newcastle Ltd., Newcastle, UK) according to the manufacturer’s protocols. In brief, formalin-fixed paraffin-embedded tumor sections were deparaffinized in xylene, rehydrated using graded ethanol, and antigen retrieval was performed in Tris-EDTA buffer (pH 9.0) containing 10 mM Tris-base, 1 mM EDTA, and 0.05% Tween-20. The slides were then incubated with peroxidase block to neutralize endogenous peroxidase and protein block to block non-specific binding, followed by incubation with the respective primary antibodies overnight at 4 °C. Subsequently, the sections were incubated with HRP polymer-conjugated secondary antibodies at room temperature for 1 h. Finally, immunoreactivity was visualized using diaminobenzidine (DAB) chromogen, and the sections were counterstained with hematoxylin, dehydrated, and mounted.

### 2.11. Western Blot Analysis

Protein lysates were extracted by RIPA buffer containing protease (Biological industries, Cromwell, CT, USA) and phosphatase (Biological industries, Cromwell, CT, USA) inhibitors. Nuclear and cytoplasmic fractions were extracted by NE-PER Nuclear and Cytoplasmic Extraction Reagents (Thermo Fisher, Pittsburgh, PA, USA) according to the manufacturer’s instructions. Protein concentration was determined using a Pierce BCA protein assay kit (Thermo Fisher, Pittsburgh, PA, USA). Equal amounts of protein samples were electrophoretically separated by sodium dodecyl sulfate polyacrylamide gel electrophoresis (SDS-PAGE) and transferred onto polyvinylidene difluoride (PVDF) membranes (Merck Millipore, Darmstadt, Germany). The membranes were blocked with 5% non-fat milk for 1 h at room temperature and then incubated with primary antibodies diluted with blocking buffer overnight at 4 °C. The next day, the membranes were washed with 0.1% TBST and subsequently incubated with HRP–conjugated secondary antibodies for an additional 1 h at room temperature. Protein bands were visualized using ECL reagents (Merck Millipore, Darmstadt, Germany) and captured by a chemiluminescence image system (UVP Inc., San Gabriel, CA, USA).

### 2.12. Animal Studies

All animal experiments were performed in accordance with the ethical guidelines approved by the Institutional Animal Care and Use Committee at National Defense Medical Center (NDMC) (Taipei, Taiwan). Male nude mice (4 weeks old) were obtained from the National Laboratory Animal Center (Taipei, Taiwan) and housed in the animal center at NDMC with free access to food and water. SAS cells (1.5 × 10^6^) transfected with scramble shRNA control (SAS-shV) or BiP shRNA (SAS-shBiP) (National RNAi Core Facility, Academia Sinica, Taipei, Taiwan) in PBS mixed with Matrigel at a 1:1 ratio were subcutaneously injected into the right flank of the mice. When the tumor size was about 100  mm^3^, the mice were randomized into two groups: one group was intraperitoneally administered with PBS, which served as a vehicle control, and the other with nicotine (1 mg/kg body weight daily) [27,28] (*n* = 5 per group). The length and width of tumors were measured every three days with Vernier calipers. The tumor volume was calculated using the following formula: volume = (length × width^2^)/2. At the end of treatment, the mice were sacrificed and the excised tumors were weighed.

### 2.13. Statistical Analysis

The Student’s *t*-test was used to determine statistically significant differences between the two groups. For more than two groups, one-way ANOVA followed by Bonferroni’s post hoc test was applied. Statistical analysis was performed and graphical representations were obtained using GraphPad Prism software version 5.01 (GraphPad Software Inc., San Diego, CA, USA). Data were represented as the means ± SEM. A *P* value less than 0.05 was regarded as being statistically significant.

## 3. Results

### 3.1. Nicotine Remarkably Induced BiP Expression in OSCC Cells

**Figure 1:**
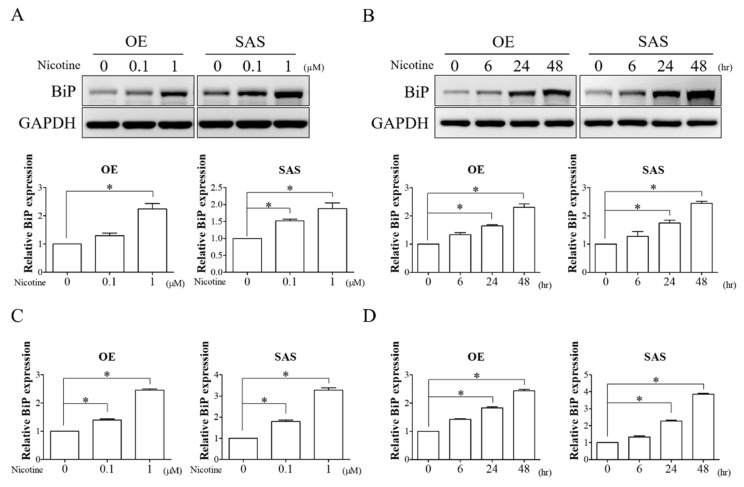
Nicotine increased BiP expression in a dose- and time-dependent manner. (**A**,**C**) OE and SAS cells were treated with 0.1 and 1 μM of nicotine for 48 h. The dose-dependent effect of nicotine on BiP expression was analyzed by Western blot analysis and quantitative RT-PCR. The graphs show the quantification of Western blots (**A**, lower panels). Band intensities were quantified using ImageJ software. The relative protein expression of BiP was normalized to GAPDH expression. (**B**,**D**) OE and SAS cells treated with 1 μM nicotine for 6, 24, and 48 h were subjected to Western blot analysis and quantitative RT-PCR for investigating the time-dependent effect of nicotine on the expression of BiP. The graphs demonstrate the quantification of Western blots (**B**, lower panels). Band intensities were analyzed using ImageJ software. The relative protein expression of BiP was normalized to GAPDH expression. GAPDH was used as the loading control. * *p* < 0.05 by one-way ANOVA followed by Bonferroni’s post hoc test. Data are presented as the mean ± SEM of three independent experiments. SEM, error bars.

Nicotine has been reported to be a crucial risk factor for oral cancer development and progression [6,7,8]. Previous studies have demonstrated that nicotine can induce the expression of BiP in cancer [19,20]. To examine the effect of nicotine on BiP expression, the expression of BiP in OSCC cells (OE and SAS) treated with various doses (0.1 and 1 μM) of nicotine for 48 h or with 1 μM nicotine for 6, 24, and 48 h was analyzed. As shown in Figure 1, protein (Figure 1A,B) and mRNA (Figure 1C,D) expressions of BiP were remarkably increased in a dose- and time-dependent manner after the cells were exposed to nicotine. These results indicated that nicotine could regulate BiP expression in OSCC cells. Since the most obvious change in BiP expression was observed in OSCC cells in response to nicotine at 1 μM for 48 h, this dose and timing of nicotine treatment were used in the following experiments.

### 3.2. BiP Was Involved in Nicotine-Stimulated Malignant Behaviors in OSCC Cells

**Figure 2:**
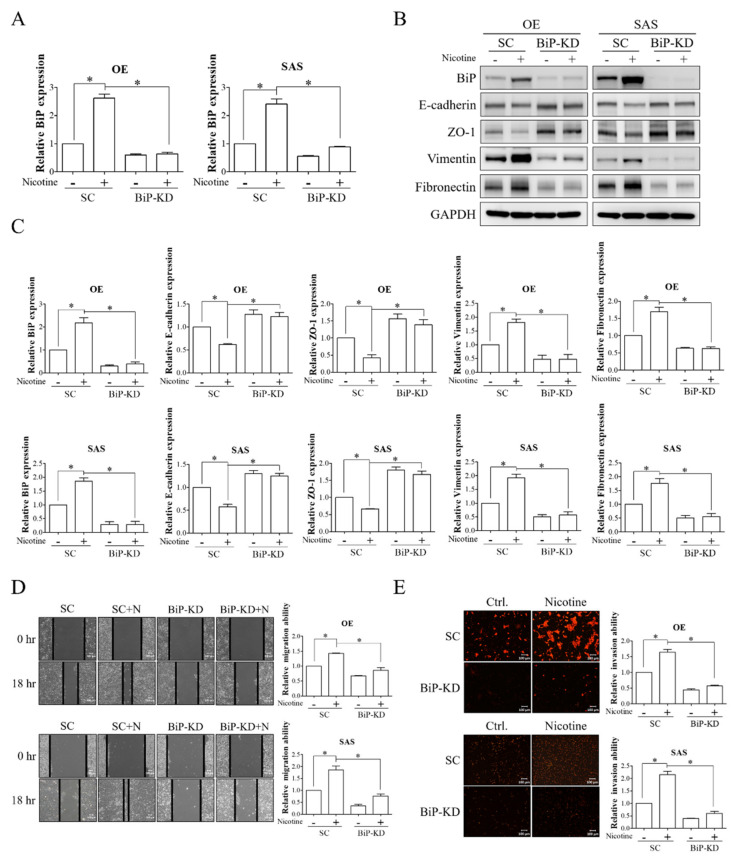
BiP was involved in the pro-malignant effect of nicotine on EMT, migration, and invasion in OSCC. OE and SAS cells were transfected with either non-targeting siRNA or BiP siRNA followed by 1 μM nicotine exposure for 48 h. (**A**) The expression of BiP was examined using quantitative RT-PCR. (**B**) The expressions of BiP, epithelial (E-cadherin and ZO-1), and mesenchymal (vimentin and fibronectin) markers in OSCC cells after the indicated treatments were analyzed by Western blot analysis. (**C**) The graphs show the quantification of Western blots. Band intensities were quantified using ImageJ software. The relative expressions of BiP, E-cadherin, ZO-1, vimentin, and fibronectin were normalized to GAPDH expression. (**D**) The ability of cell migration was investigated by wound-healing assay. Representative images were acquired, and black solid lines indicate the wound borders at 0 and 18 h post-scratching (**D**, **left** panels). The quantitative results of wound closure were determined using ImageJ software (**D**, **right** panels). The ability of migration was calculated by the area reduction at 18 h compared to the wound area at 0 h. (**E**) The invasive ability was determined by Transwell chambers pre-coated with Matrigel. Cells that penetrated across the membrane were fixed with methanol and stained with propidium iodide (PI). The quantitative results of PI-stained invasive cells were analyzed using ImageJ software (**E**, right panels). N, nicotine. SC, non-targeting siRNA-transfected cells. BiP-KD, BiP siRNA-transfected cells. GAPDH was used as the loading control. * *p* < 0.05 by one-way ANOVA followed by Bonferroni’s post hoc test. Data are presented as the mean ± SEM of three independent experiments. SEM, error bars. Scale bar, 100 μm.

The overexpression of BiP has been reported to be involved in mediating tumorigenic functions such as EMT change, migration, and invasion [29]. To explore the potential involvement of BiP in nicotine-mediated OSCC progression, OSCC cells with/without knockdown of BiP expression were exposed to nicotine, and the expressions of epithelial (E-cadherin and ZO-1) and mesenchymal (vimentin and fibronectin) markers and migratory and invasive abilities were subsequently analyzed. We observed that nicotine treatment remarkably increased BiP expression and EMT change, as demonstrated by the decreased expressions of epithelial markers (E-cadherin and ZO-1) and increased expressions of mesenchymal markers (vimentin and fibronectin), in OSCC cells (Figure 2A–C). Migration (Figure 2D) and invasion (Figure 2E) were also increased in nicotine-treated OSCC cells. Furthermore, these effects were significantly suppressed in BiP-silenced cells. These results indicated the oncogenic role of BiP in nicotine-mediated OSCC malignant behaviors.

### 3.3. Nicotine Induced BiP Expression and Tumor Progression via α7-nAChR-Akt Signaling in OSCC Cells

**Figure 3:**
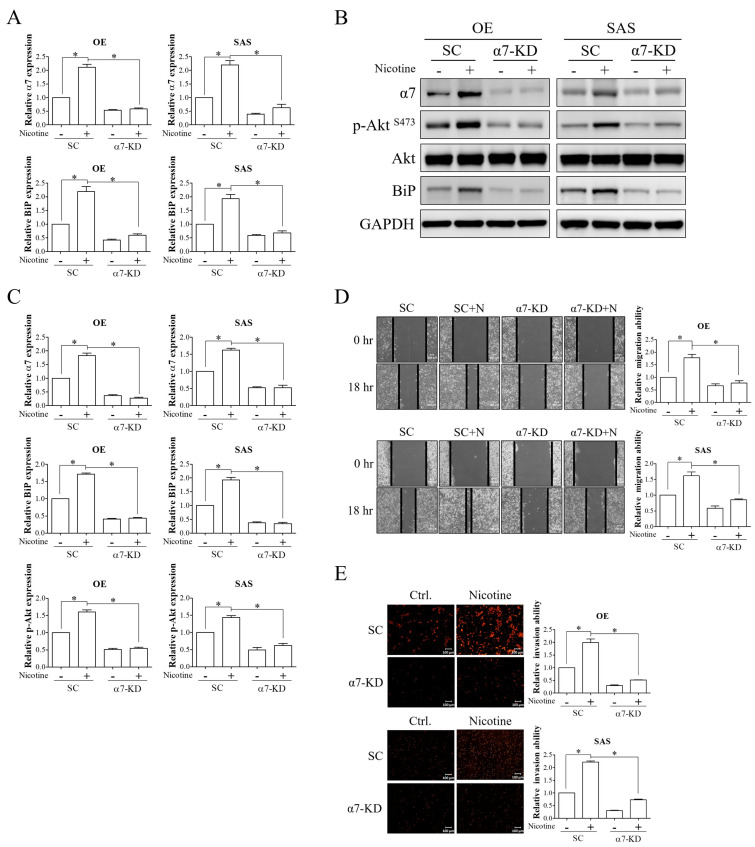
α7-nAChR-Akt signaling was involved in nicotine-induced BiP expression and malignant behaviors in OSCC. OE and SAS cells with/without α7-nAChR silencing were treated with 1 μM nicotine for 48 h. (**A**,**B**) The expressions of α7-nAChR and BiP and activation of Akt, as indicated by the expression level of phospho-Akt at Ser473, were analyzed by quantitative RT-PCR and Western blot analysis. (**C**) The graphs show the quantification of Western blots. Band intensities were quantified using ImageJ software. The relative protein expressions of α7-nAChR and BiP were normalized to GAPDH expression. The relative protein expression of phospho-Akt (Ser473) was normalized to total Akt expression. The migratory (**D**) and invasive (**E**) abilities were examined by wound healing and Transwell invasion assays. Representative images are shown, and black solid lines indicate the wound borders acquired at 0 and 18 h after scratching (**D**, **left** panels). Quantitative results of migratory cells (**D**, **right** panels) and PI-stained invasive cells (**E**, **right** panels) were determined using ImageJ software. The ability of migration was calculated by the area reduction at 18 h compared to the wound area at 0 h. N, nicotine. SC, non-targeting siRNA-transfected cells. α7-KD, α7-nAChR siRNA-transfected cells. GAPDH was used as the loading control. * *p* < 0.05 by one-way ANOVA followed by Bonferroni’s post hoc test. Data are presented as the mean ± SEM of three independent experiments. SEM, error bars. Scale bar, 100 μm.

Nicotine has been reported to promote cancer progression mainly through the activation of α7-nAChR [23]. Upon activation, α7-nAChR-mediated tumor progression has been partly attributed to the phosphoinositide 3-kinase (PI3K)/Akt signaling pathway [30]. To investigate the involvement of α7-nAChR signaling in mediating the effect of nicotine on BiP expression and malignant behaviors in OSCC, OSCC cells transfected with non-targeting siRNA or α7-nAChR siRNA were exposed to nicotine. The expression of α7-nAChR was significantly increased after nicotine treatment in scramble-transfected cells, and this effect was suppressed in α7-nAChR-silenced cells (Figure 3A–C). In addition, the stimulatory effects of nicotine on the expressions of phosphorylated Akt at serine 473 and BiP were diminished by knockdown of α7-nAChR expression (Figure 3A–C). Wound-healing migration and Transwell invasion assays showed that nicotine-induced migration and invasion were abrogated in α7-nAChR-silenced cells (Figure 3D,E). These findings indicated that α7-nAChR acted as a crucial regulator of nicotine and its effects on BiP expression and malignant behaviors in OSCC.

### 3.4. The YAP-TEAD Transcriptional Complex Was the Downstream Effector of α7-nAChR-Akt Signaling in Nicotine-Induced BiP Expression and Malignant Behaviors

**Figure 4:**
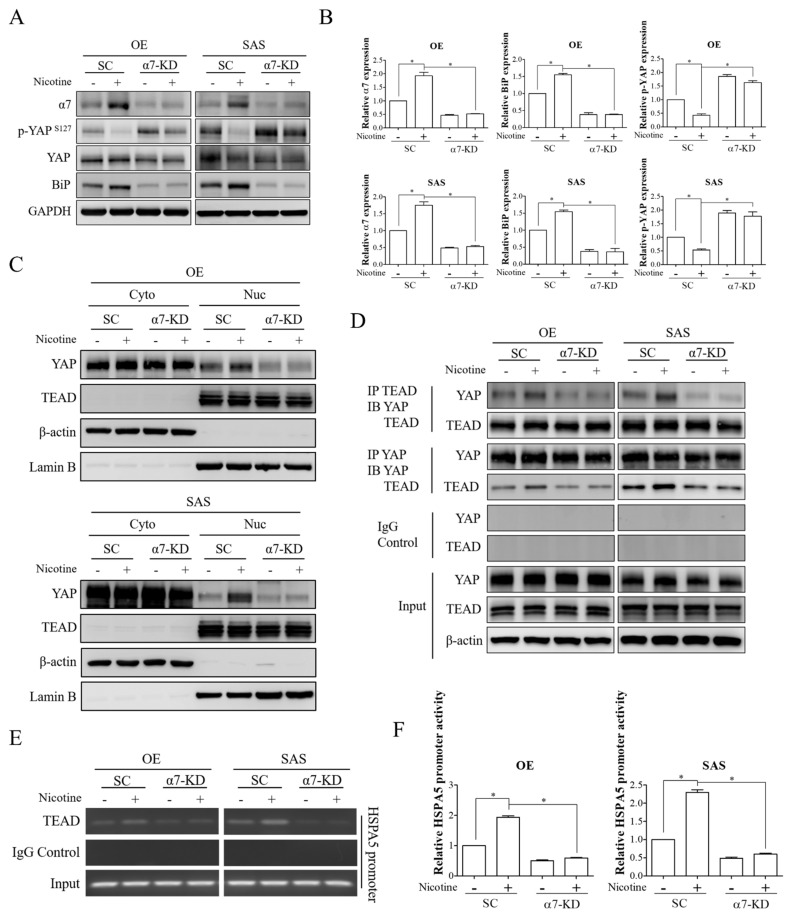
α7-nAChR-Akt signaling increased YAP activation, DNA binding, and transactivation abilities of the YAP-TEAD complex upon nicotine exposure. OE and SAS cells transfected with either non-targeting siRNA or α7-nAChR siRNA were treated with 1 μM nicotine for 48 h. (**A**) The expressions of α7-nAChR, phospho-YAP (Ser127), YAP, and BiP were assessed by Western blot analysis. (**B**) The graphs show the quantification of Western blots. Band intensities were quantified using ImageJ software. The relative expressions of α7-nAChR and BiP were normalized to GAPDH expression. The relative expression of phospho-YAP (Ser127) was normalized to total YAP expression. (**C**) Subcellular localization of YAP and TEAD were detected by investigating the expression levels of these molecules in the cytoplasmic and nuclear fractions. The cytoplasmic and nuclear extracts were obtained by NE-PER nuclear and cytoplasmic extraction reagents and investigated for YAP and TEAD expressions by Western blot analysis. β-actin and Lamin B were used as loading controls for the cytoplasmic and nuclear extracts, respectively. (**D**) The interaction of YAP with TEAD was evaluated by co-immunoprecipitation analysis. Western blot analysis of YAP and TEAD was performed after immunoprecipitation with anti-YAP, anti-TEAD, or anti-rabbit IgG antibodies. (**E**) Occupancy of TEAD on the HSPA5 promoter was analyzed by chromatin immunoprecipitation assay. Sheared chromatin was immunoprecipitated with anti-TEAD or anti-rabbit IgG antibodies followed by capture with protein agarose beads. The eluted chromatin was subjected to PCR amplification to detect DNA fragments of the HSPA5 promoter region containing the TEAD binding site. (**F**) The promoter activity of HSPA5 was examined by luciferase reporter assay. α7-nAChR-silenced OE and SAS cells were co-transfected with Cypridina luciferase reporter plasmids constructed with the HSPA5 promoter containing the TEAD binding site and red firefly luciferase plasmids, followed by treatment with 1 μM nicotine for 48 h. Luciferase activity was detected using a luciferase dual assay system. Firefly luciferase activity was used to normalize Cypridina luciferase activity. SC, non-targeting siRNA-transfected cells. α7-KD, α7-nAChR siRNA-transfected cells. GAPDH and β-actin served as the loading controls. Rabbit IgG was used as a negative control. * *p* < 0.05 by one-way ANOVA followed by Bonferroni’s post hoc test. Data are presented as the mean ± SEM of three independent experiments. SEM, error bars.

YAP transcriptional cofactor has been reported to be a potential driver of OSCC progression [31]. YAP is predominantly associated with the TEAD transcription factor to drive the transactivation of downstream target genes [32]. Regulation of YAP-TEAD transcriptional activity primarily depends on phosphorylation-dependent YAP nucleocytoplasmic shuttling [32]. When phosphorylated by upstream kinases, YAP is localized in the cytoplasm and is unable to interact with TEAD. In contrast, upon dephosphorylation, YAP can translocate into the nucleus and form a complex with TEAD. In addition, a previous study has demonstrated that nicotine can induce nuclear localization and activation of YAP [33]. Hence, to evaluate the potential role of the YAP-TEAD complex as a downstream regulator of the α7-nAChR-Akt pathway in nicotine-induced BiP expression, OSCC cells with/without depletion of α7-nAChR expression were treated with nicotine, and the phosphorylation status of YAP was subsequently detected. Nicotine exposure resulted in dephosphorylation of YAP in OSCC cells, as evidenced by a decreased level of phosphorylated YAP at serine 127, and the effect of nicotine on YAP dephosphorylation was diminished in α7-nAChR-silenced cells (Figure 4A,B). Since YAP dephosphorylation leads to its nuclear accumulation, the role of α7-nAChR in the subcellular distribution of YAP after nicotine treatment in OSCC cells was evaluated. Increased nuclear translocation of YAP was observed in the cells treated with nicotine, and this was suppressed by silencing α7-nAChR expression (Figure 4C). Furthermore, we observed that nicotine treatment resulted in increased interaction between YAP and TEAD, but that this interaction was decreased in α7-nAChR-knockdown OSCC cells after nicotine exposure (Figure 4D). These findings indicated that α7-nAChR signaling was the crucial regulator of nicotine-induced YAP nuclear distribution and activation.

The TEAD protein has been shown to have an N-terminal DNA binding domain which is responsible for recognizing the sequence motif 5′-GGAATG-3′ [34]. We observed one putative TEAD binding site located within the promoter region of HSPA5, which was the gene that encoded BiP. Therefore, we investigated the association between TEAD and the HSPA5 promoter. We observed that the occupancy of TEAD on the HSPA5 promoter was remarkably increased in nicotine-treated cells. Moreover, this binding was disrupted by knockdown of α7-nAChR expression (Figure 4E). Owing to the stronger association between TEAD and the HSPA5 promoter region after nicotine treatment, we examined the promoter activity of HSPA5. Nicotine treatment markedly led to elevated promoter activity of HSPA5, and this was decreased by silencing α7-nAChR expression (Figure 4F). These results indicated that nicotine modulated the DNA binding and transactivation abilities of the YAP-TEAD complex through α7-nAChR signaling in OSCC.

**Figure 5:**
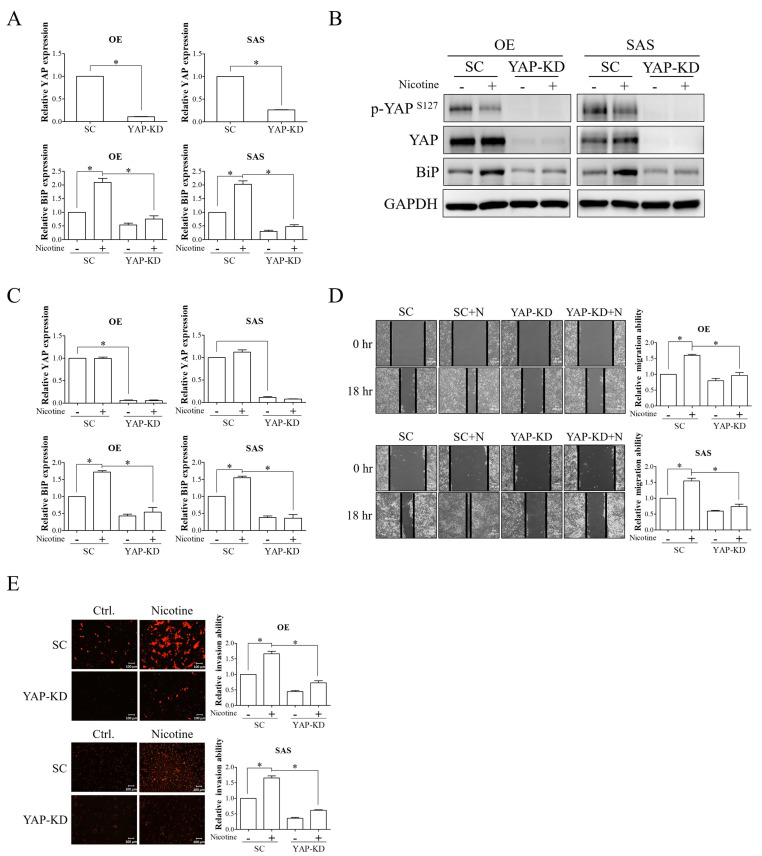
The YAP-TEAD transcriptional complex was involved in the regulation of nicotine-induced BiP expression and tumor malignancy in OSCC. OE and SAS cells transfected with non-targeting siRNA or YAP siRNA were exposed to 1 μM nicotine for 48 h. (**A**,**B**) The expressions of phospho-YAP (Ser127), YAP, and BiP were analyzed by quantitative RT-PCR and Western blot analysis. (**C**) The graphs show the quantification of Western blots. Band intensities were quantified using ImageJ software. The relative protein expressions of BiP and YAP were normalized to GAPDH expression. The abilities of cell migration (**D**) and invasion (**E**) were investigated using wound-healing and Transwell invasion assays, respectively. Black solid lines on the acquired images indicate the wound borders at 0 and 18 h post-scratching (**D**, **left** panels). Quantitative results of migratory cells across the wound borders (**D**, **right** panels) and PI-stained invasive cells (**E**, **right** panels) were determined using ImageJ software. The migratory ability was calculated by the area reduction at 18 h compared to the wound area at 0 h. *N*, nicotine. SC, non-targeting siRNA-transfected cells. YAP-KD, YAP siRNA-transfected cells. GAPDH served as the loading control. * *p* < 0.05 by Student’s *t*-test or one-way ANOVA followed by Bonferroni’s post hoc test. Data are presented as the mean ± SEM of three independent experiments. SEM, error bars. Scale bar, 100 μm.

We subsequently investigated whether the YAP-TEAD complex was a downstream effector of α7-nAChR signaling in nicotine-induced BiP expression by knockdown of YAP expression. The mRNA and protein expressions of YAP were significantly decreased in OE and SAS cells transfected with YAP siRNA compared to those with non-targeting siRNA (Figure 5A–C). The effect of nicotine on the induction of BiP expression was abolished by knockdown of YAP expression (Figure 5A–C). Using wound-healing migration and Transwell invasion assays, we demonstrated that YAP silencing resulted in decreased nicotine-induced migration and invasion in OSCC cells (Figure 5D,E). These results indicated that the YAP-TEAD transcriptional complex was a downstream regulator of α7-nAChR signaling in nicotine-stimulated BiP expression and tumor malignancy.

### 3.5. BiP Silencing Decreased Nicotine-Induced Cell Growth and EMT in Tumor-Bearing Mice

**Figure 6:**
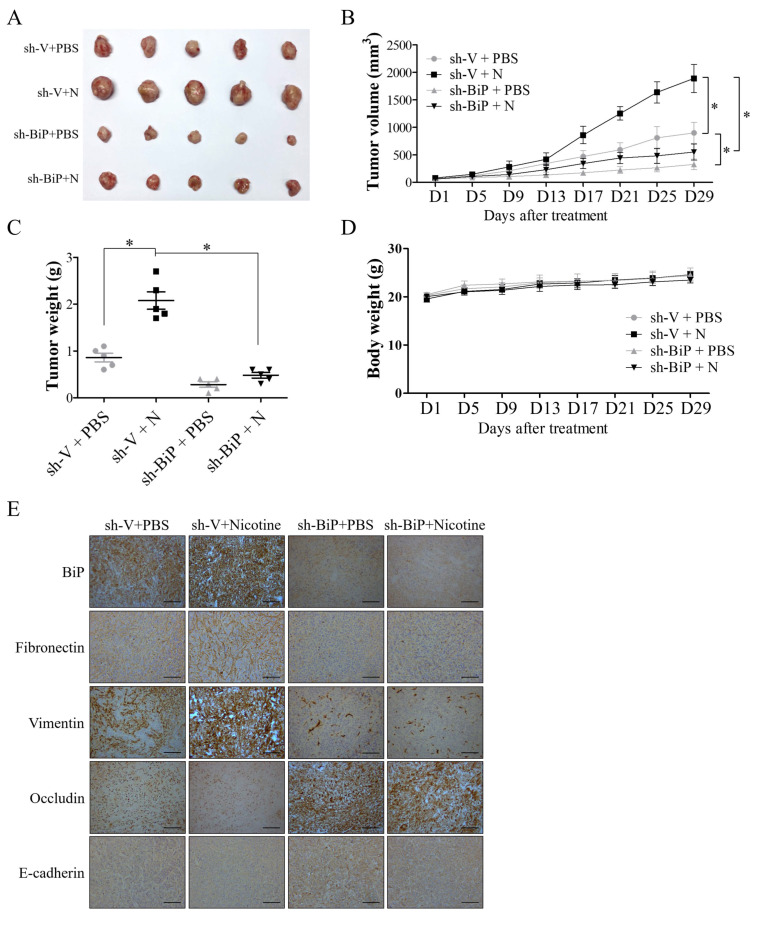
BiP inhibition suppressed nicotine-induced oral cancer progression in nude mice. SAS cells transfected with scramble shRNA control (SAS-shV) or BiP shRNA (SAS-shBiP) (1.5 × 10^6^ cells/mice) were subcutaneously implanted into the right flank of nude mice. When the tumor size was about 100 mm^3^, the tumor-bearing mice were given daily intraperitoneal injections of either PBS or 1 mg/kg nicotine for one month. Following treatment, the mice were sacrificed and the tumor tissues were subjected to immunohistochemical staining for BiP and epithelial–mesenchymal transition (EMT) markers. (**A**) Representative images of dissected tumors (*n* = 5) from the nude mice. Average tumor growth curve (**B**) and tumor weight (**C**) in each group of mice (*n* = 5) were also recorded during treatment and at the time of mice sacrifice, respectively. The xenograft tumor volumes were measured twice every week using Vernier calipers and calculated using the formula: volume = (length × width^2^)/2. (**D**) The average body weight change of the nude mice. Bodyweight was measured twice every week. (**E**) Representative immunohistochemical staining images of the expressions of BiP, mesenchymal (fibronectin and vimentin), and epithelial (occludin and E-cadherin) markers in tumor tissues. N, nicotine. * *p* < 0.05 by one-way ANOVA followed by Bonferroni’s post hoc test. Data are presented as the mean ± SEM of 5 mice in each group. SEM, error bars. Scale bar, 100 μm.

To further validate the involvement of BiP in nicotine-induced OSCC progression, nude mice subcutaneously implanted with SAS-shV or SAS-shBiP cells were subjected to PBS or nicotine treatment. Tumor growth and size were remarkably increased in the nicotine-treated group compared with the PBS-treated group, whereas BiP silencing inhibited nicotine-induced cell growth (Figure 6A–C). Body weight loss was not observed in all treatment groups (Figure 6D). Furthermore, immunohistochemical results showed increased expressions of BiP and mesenchymal markers (fibronectin and vimentin) and decreased expressions of epithelial markers (occludin and E-cadherin) in tumors from the mice treated with nicotine (Figure 6E). Moreover, the effect of nicotine on EMT change was suppressed by BiP inhibition (Figure 6E). These results further indicated the pro-tumor role of BiP in nicotine-mediated OSCC malignancy in mice.

## 4. Discussion

Cigarette smoking is highly associated with the development of oral cancer, accounting for 75% of all oral cancer cases [35]. Nicotine is an important component in cigarette smoking, and it has been reported to cause the malignant behaviors of OSCC [6,7,8]; however, the molecular mechanisms underlying these effects have not been fully elucidated. In particular, the effect of nicotine on the ER stress-responsive protein, BiP, has not been reported in OSCC. Our study demonstrated that BiP was involved in nicotine-induced OSCC progression. Notably, the underlying mechanisms of nicotine-induced BiP expression were through α7-nAChR-Akt signaling and the subsequent activation of the YAP-TEAD transcriptional complex.

In this study, we found that α7-nAChR participated in the nicotine-induced expression of BiP. Homomeric α7-nAChR is ubiquitously expressed in mammalian cells and is regarded to be the principal receptor involved in nicotine-mediated cancer progression [36]. Our results showed that α7-nAChR expression was upregulated in OSCC cells in response to nicotine treatment. Consistently, an increased expression of α7-nAChR has been observed in normal human bronchial epithelial cells (NHBE) upon nicotine exposure [37]. Schaal et al. reported that nicotine could induce the expression of α7-nAChR through the activation of α7-nAChR signaling [38]. Moreover, they reported that the treatment of A549 non-small cell lung adenocarcinoma cells with α-bungarotoxin (α-BTX), an inhibitor of α7-nAChR, abrogated the nicotine-mediated induction of α7-nAChR [38]. These results indicate the existence of an autoregulatory feed-forward loop in nicotine-induced α7-nAChR upregulation, which can further amplify the downstream signals mediated by nicotine. Hence, it has been suggested that antagonists of α7-nAChR may have anti-tumor effects against tobacco-related cancers [39,40]. For example, Dinicola et al. reported the effect of nicotine on interfering with the cytotoxicity of chemotherapeutics was suppressed by exposure to α-BTX [39]. Furthermore, genetic or pharmacological blockade of α7-nAChR has been shown to abolish nicotine-induced tumor growth in non-small cell lung cancer [40]. In the present study, we showed that nicotine could increase the expression of the ER stress-responsive protein, BiP, through α7-nAChR signaling. The controversial link between nAChR signaling and UPR components has been previously reported in non-tumor cells [26,41]. For example, Srinivasan et al. showed that pharmacological chaperoning of nAChR after nicotine treatment could repress ER stress and UPR activation, which in turn resulted in neuroprotection of Neuro-2a cells [41]. In addition, Ishibashi et al. observed that nicotine suppressed ER stress-induced apoptosis in pancreatic β-cells by regulating UPR activation through α7-nAChR [26]. The detailed mechanisms contributing to this discrepancy have not been well evaluated, and further studies are needed to investigate the role of nAChR in context-specific regulation of UPR signaling upon nicotine treatment in different tissues.

Our results further demonstrated that nicotine regulated the DNA binding ability and activity of the YAP-TEAD transcriptional complex through α7-nAChR-Akt signaling. The YAP-TEAD complex is normally involved in the regulation of cell growth, proliferation, and organ development [42]. However, the overexpression of YAP has been observed in multiple types of cancer, including OSCC, liver cancer, colon cancer, and lung cancer [43]. In addition, YAP has been reported to be a crucial driver of cancer cell proliferation, invasion, migration, metastasis, and resistance to anti-cancer therapies [44]. Clinically, YAP expression has been shown to be significantly higher in OSCC tissues compared to adjacent normal tissues, and increased nuclear-diffused staining of YAP has been significantly associated with poor differentiation in OSCC tissues [45]. Furthermore, the expression of YAP has been positively correlated with smoking status in patients with esophageal squamous carcinoma [33]. Several oncogenes have been reported to be the downstream targets of YAP by interacting with TEAD. For example, vimentin has been shown to be a direct target of the YAP-TEAD complex, which is an intermediate filament protein that plays a role in cell motility and adhesion during EMT [46]. In addition, the YAP-TEAD complex has been demonstrated to increase the expression of the anti-apoptotic molecule, Bcl-2, leading to resistance to cell apoptosis in OSCC [45]. Notably, our results showed that nicotine could induce BiP expression via activation of YAP, and that knockdown of YAP expression diminished the nicotine-stimulated expression of BiP in OSCC. Therefore, our findings provide evidence that the YAP-TEAD complex functions as a downstream effector of α7-nAChR signaling, and that this is involved in the nicotine-induced expression of BiP and tumor progression in OSCC.

Previous studies have reported controversial results regarding the role of BiP in OSCC progression. BiP has been positively associated with OSCC malignant behaviors [17,18]. For example, BiP has been shown to be overexpressed in oral cancer cell lines compared to normal oral keratinocytes. In addition, knockdown of BiP has been shown to remarkably decrease cell growth, migratory and invasive abilities [17]. Xia et al. reported that OSCC tissues showed significantly higher expressions of BiP than normal oral tissues and that the expression of BiP was positively associated with clinicopathological parameters, including tumor size, pathological stage, histological grade, lymphatic metastasis, and distant metastasis in OSCC patients [18]. On the other hand, Huang et al. showed that weakly expressed BiP was significantly correlated with tumor-node-metastasis (TNM) stage and neck lymph node metastasis in patients with OSCC [47]. These contradictory findings regarding the role of BiP in OSCC may be explained by differences in subcellular localization and time-dependent expression of BiP [48]. Recent evidence has shown that BiP is typically localized in ER, but that in some circumstances BiP can also be secreted or relocate to outside the cytoplasm, nucleus, mitochondria, and cell membrane [49]. Inflammatory cytokines and the ER stress inducer, thapsigargin, have been shown to induce BiP translocation to the plasma membrane in pancreatic β-cells and human embryo kidney fibroblast cells, respectively [50,51]. The overexpression of BiP has also been shown to promote the relocation of BiP to the cell surface in the absence of ER stress [51]. Anti-cancer drugs such as the pan-Bcl-2 inhibitor obatoclax (OBX) have been shown to strongly stimulate BiP translocation to the cell membrane in multiple myeloma (MM) cells [52]. In addition, the nuclear localization of BiP has been shown to be markedly higher in lung adenocarcinoma compared with normal lung tissues [53]. Notably, cigarette smoking may also influence the subcellular localization of BiP, as a previous study has reported increased secretion of BiP into bronchoalveolar lavage fluid (BALF) in chronic cigarette smokers [54]. We also noted that BiP can also translocate to the cell surface and the nucleus in OSCC tissues from mice (Figure S1). Depending on the different subcellular compartments of BiP, there may be diverse induction of intracellular signaling pathways and stimulation of pro-apoptotic or pro-survival responses in cancer. For example, cell surface BiP acts as a multifunctional receptor, and it has been demonstrated to be involved in the stimulation of cell proliferation, angiogenesis, and therapeutic resistance via activation of PI3K/Akt signaling in cancer [55]. In addition, oral cancer cells expressing cell surface BiP have been shown to have cancer stemness properties and radioresistance [56]. Conversely, interactions of cell surface BiP with ligands, Kringle 5 and Par-4, have been shown to potentially lead to activation of pro-apoptotic pathways in tumors and endothelial cells [55]. Kern et al. reported that the secretion of BiP conferred drug resistance in myeloma and endothelial cells and increased cell proliferation of colon cancer cells [57,58]. Furthermore, the kinetics of BiP expression may also affect the role of this molecule in cancer. In esophageal cancer, a strong BiP expression has been observed in the early and advanced stage rather than the middle stage [59]. In the present study, our findings provide scientific evidence that BiP plays an oncogenic role in mediating the malignant behaviors of OSCC after nicotine exposure. However, to clarify the role of BiP in OSCC upon nicotine treatment, further investigations of the subcellular distribution and dynamic expression of BiP are needed.

**Figure 7:**
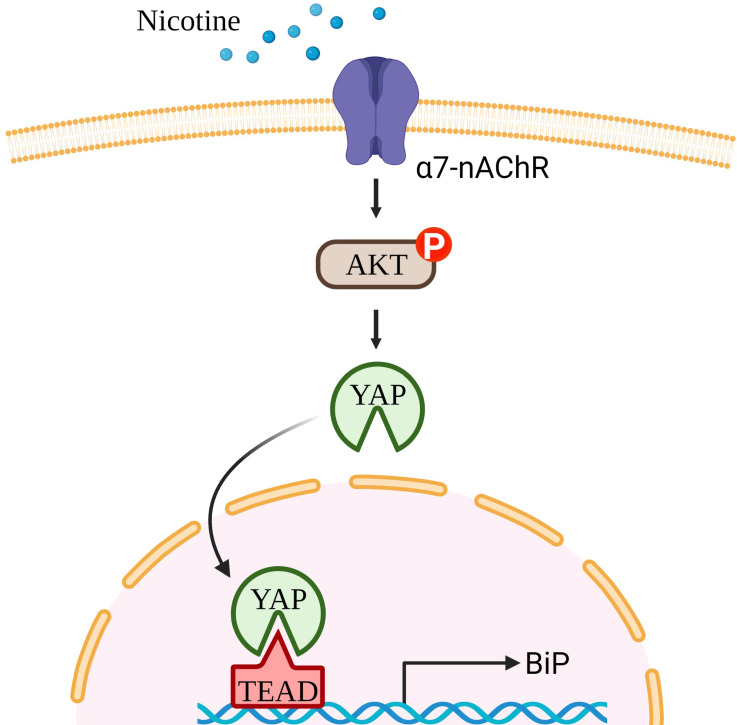
Nicotine stimulated OSCC malignancy through YAP-dependent BiP induction. Nicotine promoted malignant behaviors of OSCC, including EMT change, migration, and invasion, via inducing BiP expression. Mechanistically, nicotine increased BiP expression through α7-nAChR-Akt signaling and subsequent YAP dephosphorylation and nuclear translocation. Nuclear YAP together with TEAD increased HSPA5 promoter activity in OSCC cells after nicotine treatment.

In conclusion, our study showed that nicotine increased the malignant behaviors of OSCC cells, including EMT change, migratory and invasive abilities, by upregulating BiP expression (Figure 7). Mechanistically, the effect of nicotine on the stimulation of BiP expression was through α7-nAChR-Akt signaling, followed by activation of the YAP-TEAD transcriptional complex. These findings provide a novel insight into the pathophysiological mechanisms of nicotine-induced oral cancer progression. Furthermore, our results may provide a potential therapeutic target molecule in nicotine-medicated oral cancer malignancy, which may improve the therapeutic outcomes of patients with tobacco-associated cancers.

## Figures and Tables

**Table 1:** The primer sequences used for quantitative RT-PCR.

Gene	5′-3′	Sequences
BiP	Forward	TGA CAT TGA AGA CTT CAA AGC T
	Reverse	CTG CTG TAT CCT CTT CAC CAG T
α7-nAchR	Forward	GCT GGT CAA GAA CTA CAA TCC C
	Reverse	CTC ATC CAC GTC CAT GAT CTG
YAP	Forward	TGA ACA AAC GTC CAG CAA GAT AC
	Reverse	CAG CCC CCA AAA TGA ACA GTA G
GAPDH	Forward	CCA CAT CGC TCA GAC ACC AT
	Reverse	TGA CCA GGC GCC CAA TA

## Data Availability

Not applicable.

## Supplementary Materials

The following are available online at https://www.mdpi.com/article/10.3390/cells10082080/s1, Figure S1: Subcellular distribution of BiP in OSCC tissues from mice.
