# Supplementary material for: YAP-Dependent BiP Induction Is Involved in Nicotine-Mediated Oral Cancer Malignancy
Source: Cells. 2021 Aug 13;10(8):2080. doi: 10.3390/cells10082080 (PMC8392082; doi:10.3390/cells10082080)
Supplement: Supplementary file 1 [file cells-10-02080-s001.zip › cells-1312268-supplementary.pdf]

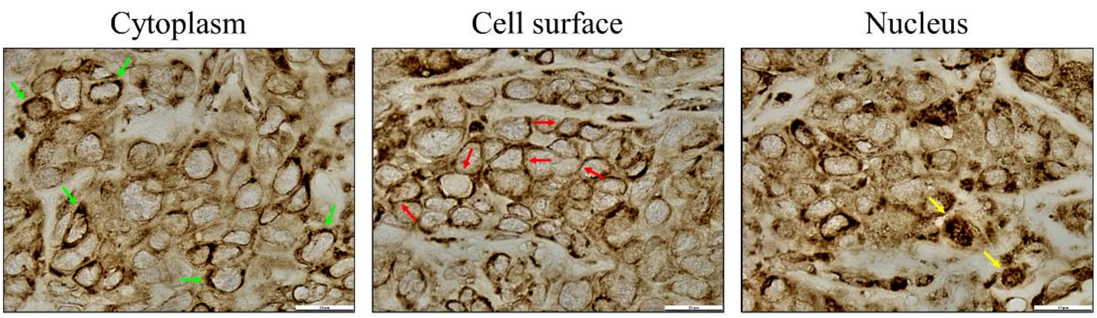

Figure S1. BiP was located in the cytoplasm, the cell surface, and the nucleus of OSCC tissues from tumor-bearing mice. Representative immunohistochemical images for BiP staining in OSCC tissues are shown. Green arrows indicate cytoplasmic localization of BiP. Red arrows indicate cell surface localization of BiP. Yellow arrows indicate nuclear localization of BiP. Scale bar, 20 μm.
